# Covert Communication with Cognitive Jammer Embedded in Public Communication System

**DOI:** 10.3390/s26082423

**Published:** 2026-04-15

**Authors:** Xiangyu Zhao, Yinjie Su, Zhuxian Lian, Yajun Wang, Zhibin Xie

**Affiliations:** School of Oceanography, Jiangsu University of Science and Technology, Zhenjiang 212000, China; 17826600006@163.com (X.Z.); zhuxianlian@just.edu.cn (Z.L.); wangyj1859@just.edu.cn (Y.W.); xiezhibin@just.edu.cn (Z.X.)

**Keywords:** covert communication, cognitive jammer, detection error, average covert rate

## Abstract

In this work, we investigate covert communication with a cognitive jammer (CJ) embedded in a public communication system. Specifically, while the transmitter continuously transmits public messages to the receiver, it may also transmit its covert messages opportunistically, and a CJ is utilized to assist the covert communication. To evaluate the covert performance, we derive the detection error probability (DEP) for both the CJ and the warden, as well as the average covert rate (ACR). Our analysis indicates that applying a CJ to assist covert communication embedded in a public communication system can outperform the scheme with an uninformed jammer or without a jammer in terms of covertness. A high public transmission rate can degrade the covertness; however, improving the detection accuracy of the CJ can contribute to enhanced performance in terms of both covertness and ACR.

## 1. Introduction

Owing to the broadcast characteristics of wireless media, wireless communication offers great convenience, while simultaneously introducing significant security vulnerabilities. Although conventional encryption techniques protect communication contents, they do not conceal the existence of the communication itself. In sensitive scenarios, such as military operations and private Internet of Things (IoT) data transmission, the exposure of communication activities can lead to severe consequences. Covert communication, also referred to as low probability of detection (LPD) communication, has therefore arisen to tackle this problem. Its core objective is to shield communication activities from detection, even against proactive wardens, and thus it attains a higher level of security.

The theoretical foundation of covert communication was pioneeringly established by Bash et al. Considering additive white Gaussian noise (AWGN) channels, the authors in [1,2] established the square root law, i.e., a maximum of $O(\sqrt{n})$ bits can be transmitted reliably over *n* channel uses. As *n* approaches infinity, the covert rate diminishes to zero. Then, the authors in [3] revealed that positive-rate reliable covert communication remains achievable when the warden exhibits noise uncertainty.

Based on this pioneering research, significant research efforts in the field of covert communication have been witnessed. Covert communication has recently been integrated with techniques such as unmanned aerial vehicles (UAVs), reconfigurable intelligent surfaces (RISs), integrated sensing and communication (ISAC), and multiple-input multiple-output (MIMO) [4]. Recently, some studies have focused on covert communication enabled by UAVs. The authors in [5] investigated covert communication in UAV-assisted IoT systems, where the transmit power and hovering location of the UAV were optimized to maximize the signal-to-noise ratio (SNR) of the legitimate receiver. The authors in [6] proposed a finite-blocklength covert autonomous aerial vehicle (AAV)-relaying scheme, where the effective covert throughput was maximized through joint optimization of the blocklength, transmit power, and hovering location of the AAV. In [7], the authors investigated a UAV-assisted covert communication scenario, where an artificial potential field (APF)-based approach was proposed to obtain the closed-form expression of the optimal continuous trajectory of the UAV. The authors in [8] investigated the covertness and reliability of a friendly jammer-aided UAV relay system under co-channel interference (CCI). The authors in [9] investigated covert communication in a UAV-enabled network serving clustered ground devices. The authors in [10] investigated UAV-enabled covert communication with a movable antenna against cooperative detection by multiple wardens, where the segmentation ratio, transmit beamforming, trajectory, and antenna positions were jointly optimized to maximize the minimum throughput among users under covertness constraints.

RIS-assisted covert communication has also attracted the attentions of researchers. In [11], the authors investigated a multi-user covert communication scenario, and it was demonstrated that an RIS-assisted scheme can enhance the performance of covert communication. The authors in [12] investigated covert communication with aerial RIS, and successive convex approximation (SCA) and semidefinite relaxation (SDR) techniques were adopted to address the problem of minimizing the average covert age of information (CAoI). A dual-functional RIS-assisted cooperative relaying scheme was proposed in [13], where a block coordinated ascent (BCA)-based iterative algorithm was developed to jointly optimize the power allocation coefficients to maximize the system security probability (SSP). The authors in [14] investigated an RIS-assisted integrated sensing and covert communication system with finite blocklength, considering both non-adaptive and adaptive RIS operation modes.

ISAC can provide sensing and communication services simultaneously, and it has been considered in the scenario of covert communication. A joint optimization problem for covert communication in an ISAC network was investigated in [15], where a block coordinate descent (BCD)-based algorithm was developed to alternately optimize the beamforming vectors and the UAV’s trajectory. In [16], the authors investigated covert communication with ISAC in the presence of multiple wardens, and an SCA-based algorithm was developed to achieve robust beamforming. An ISAC system with a simultaneously transmitting and reflecting reconfigurable intelligent surface (STAR-RIS) was investigated in [17], where the transmit beamforming at the base station and the beamforming at the STAR-RIS were jointly optimized to maximize the covert rate. The authors in [18] investigated a secure full-duplex ISAC system, in which the base station senses the physical parameters of eavesdroppers while ensuring uplink and downlink secrecy rates. A power-efficient secure ISAC optimization framework and a solution algorithm were proposed.

Recently, substantial research efforts were devoted to jammer-assisted covert communication. The authors in [19] proved that an uninformed jammer can help the transmitter to achieve a positive rate in AWGN and block fading channels. Covert communication with a wireless-powered jammer was investigated in [20], where the jammer can harvest energy from the receiver and generate jamming signals to confuse the warden. The authors in [21] investigated covert communication assisted by an energy-harvesting jammer for a multi-relay IoT system, and a quality-of-service (QoS)-aware relay and jammer selection scheme was proposed. A two-phase covert communication system with RIS and wireless-powered jammer was investigated in [22], where the effective transmission rate was improved by optimizing the phase switching ratio. The authors in [23] proposed an energy-efficient covert communication scheme with a probabilistic jammer, where the jamming probability and transmit power were optimized to maximize the energy efficiency. In [24], the authors demonstrated that probabilistic jamming can achieve better performance than continuous jamming under the same covertness and average jamming power constraints. To address the challenge that suspicious users are located far from the monitor, making proactive eavesdropping difficult, the authors in [25] investigated a cooperative cognitive radio (CR) network to facilitate distant eavesdropping. A covert communication network with spatially stochastic distributed jammers was studied in [26], where the spatial distribution of the jammers was modeled as a β-Ginibre point process. In [27], the authors investigated a multi-node cooperative covert communication network, where a jammer selection scheme was proposed to degrade the detection accuracy of the warden. A cooperative deception strategy in the presence of a multi-antenna adversary was proposed in [28]. It is demonstrated that the number of antennas can have a positive effect on the covert rate for various channel state information (CSI) scenarios. The authors in [29] investigated covert communication with multiple jammers, where the transmission rate can be improved through jammer selection and power control.

In contrast to traditional jammer that continuously transmits jamming signals, a cognitive jammer (CJ) only transmits jamming signals when it detects that no covert transmission exists. Thus, it can avoid allowing the warden to gather more information about the jamming signal over prolonged observation and reduce the probability of being deciphered by the warden. The authors in [30] investigated covert communication assisted by a CJ, which is equipped with a matched filter. The prerequisite for the CJ’s detection is that it knows the codebook used by the transmitter and the instantaneous CSI of all the links. The authors in [31] investigated finite block-length covert communication in a dual-hop space–air–ground integrated network. The prerequisite for the CJ’s detection is also that it knows the instantaneous CSI of all the links.

In this paper, we consider a commonly used scenario, i.e., covert communication embedded in a public communication system. Specifically, a transmitter transmits public messages to a receiver while also opportunistically transmitting its covert messages, and a CJ is used for disrupting the detection of the warden. In our considered scenario, to ensure the successful transmission of public messages, a fixed-rate transmission scheme was adopted. The transmit power of the transmitter depends on the instantaneous CSI of the link for public messages, which cannot be obtained by the CJ and the warden, and thus leads to channel uncertainty for the detection. Therefore, the detection methods adopted by the CJ in [30,31] cannot be directly applied to the considered scenario. Instead, we adopted a detection method based on energy detection. Since the channel uncertainty can reduce the detection accuracy of both the CJ and the warden, and thereby may affect the performance of covert communication, we aim to reveal the performance behavior of covert communication in the considered scenario. To the best of our knowledge, this is the first research to investigate covert communication that is assisted by a CJ and embedded in a public communication system where channel uncertainty exists. We derive the DEP for both the CJ and the warden, as well as the expression for the average covert rate (ACR). Further, the effects of system parameters on the performance of covert communication are analyzed. Our research reveals that even though channel uncertainty impairs the detection performance of the CJ and thus has a negative impact on the covert performance, the application of a CJ can still effectively enhance the covert performance of the system compared to the schemes with an uninformed jammer (i.e., a jammer that continuously transmits jamming signals) and without a jammer. Moreover, increasing jamming power can disrupt the detection of the warden; however, it may lead to a decrease in ACR. A high public transmission rate can degrade the covertness; however, improving the detection accuracy of the CJ can contribute to enhancing performance in terms of both covertness and ACR.

## 2. Materials and Methods

### 2.1. System Model

**Figure 1:**
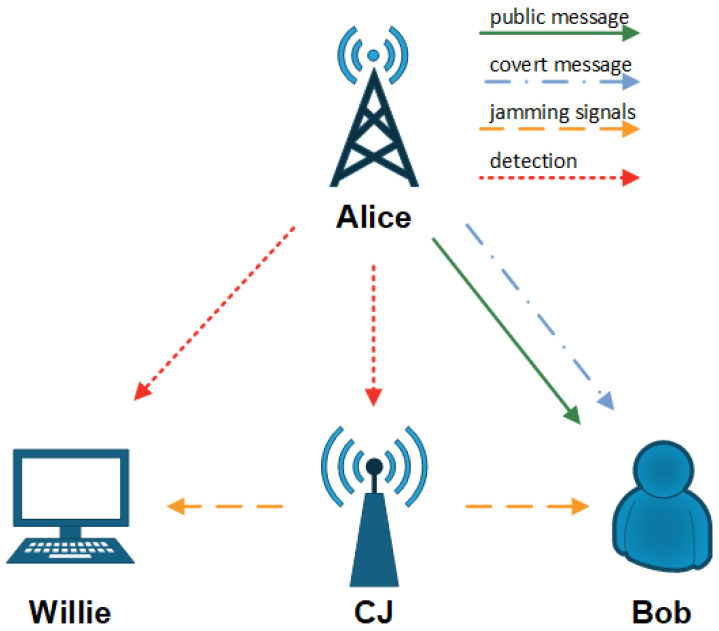
System model of covert communication with a cognitive jammer.

We consider a covert communication system comprising four nodes, each equipped with a single antenna, namely a transmitter (Alice), a receiver (Bob), a warden (Willie), and a CJ. The system model is illustrated in Figure 1. Alice transmits public messages to Bob while opportunistically transmitting covert messages. To ensure successful transmission, fixed-rate transmission is adopted for the public messages. The covert messages are transmitted with a power $P_{c}$, and the CJ generates timely jamming signals to disrupt the detection of Willie. The channel for Alice-to-Bob, Alice-to-Willie, Alice-to-CJ, CJ-to-Bob, and CJ-to-Willie links are denoted by $h_{ab}$, $h_{aw}$, $h_{aj}$, $h_{jb}$, and $h_{jw}$, respectively. All links are modeled as undergoing quasi-static Rayleigh fading. The channel coefficients are constant with one time slot and vary independently from one time slot to another. The channel coefficients follow the Rayleigh distribution, where $h_{pq}$∼$\mathrm{CN}(0,\lambda_{pq})$, $pq\in \left\{ab,aw,aj,jb,jw\right\}$, and $\lambda_{pq}$ denotes the large-scale path loss of the wireless channel. Unlike continuous jamming strategies, the CJ decides to transmit jamming signals based on its own detection results. Specifically, for each time slot, the work of the CJ consists of two phases. In the first phase, with duration $T_{J}$, the CJ detects whether Alice transmits its covert messages. In the second phase, with duration $T_{S}$, the CJ transmits its jamming signals or not according to its prior detection result. Each time slot, with duration *T*, consists of *n* channel uses, and $T_{J}=T_{S}=T/2$. It is assumed that the CJ periodically receives pilot symbols from Alice and Bob to obtain the instantaneous CSI of its direct links (Alice-to-CJ and CJ-to-Bob) through channel estimation techniques. Meanwhile, the warden, Willie, periodically receives pilot symbols from Alice and CJ, and thus it is capable of obtaining the instantaneous CSI of its direct links (Alice to Willie and CJ to Willie) through channel estimation techniques.

In the considered system, when Alice transmits only public messages to Bob without sending covert messages, the received signal at Bob can be given by

(1) $$y_{b}\left[i\right]=\sqrt{P_{r,0}}h_{ab}x_{p}\left[i\right]+\delta \sqrt{P_{j}}h_{jb}x_{j}\left[i\right]+n_{b}\left[i\right],$$

where $P_{r,0}$ denotes the transmit power for public messages, and $P_{j}$ denotes the transmit power of CJ; $x_{p}\left[i\right]$ and $x_{j}\left[i\right]$ denote the transmit signal for public messages and jamming signals, respectively; $n_{b}\left[i\right]$∼$\mathrm{CN}(0,\sigma_{b}^{2})$ denotes the AWGN at Bob, and *i* is the channel use index; and δ is an indicator variable, i.e., δ=1 indicates the presence of jamming and δ=0 indicates the absence of jamming. The received signal-to-interference-plus-noise ratio (SINR) at Bob can be given by

(2) $$\begin{array}{ccc} {\mathrm{SIN}R}_{b} & = & \frac{P_{r,0}{\left|h_{ab}\right|}^{2}}{\delta T_{S}/TP_{j}{\left|h_{jb}\right|}^{2}+\sigma_{b}^{2}}\\ & = & \frac{2P_{r,0}{\left|h_{ab}\right|}^{2}}{\delta P_{j}{\left|h_{jb}\right|}^{2}+2\sigma_{b}^{2}}. \end{array}$$

Adopting fixed-rate transmission, Alice transmits public messages to Bob with a fixed rate $r_{sd}$. In order to ensure successful decoding, even in the presence of jamming, the received SINR at Bob should meet the threshold $\gamma_{th}=2^{r_{sd}}-1$. Therefore, $P_{r,0}$ can be given by

(3) $$P_{r,0}=\frac{\gamma_{th}\left(P_{j}{|h_{jb}|}^{2}+2\sigma_{b}^{2}\right)}{2{\left|h_{ab}\right|}^{2}}.$$

Considering the maximum power constraint at Alice, i.e., $P_{r,0}\leq P_{\max}$, the necessary condition for Alice to perform its public message transmission can be given by

(4) $$A=\left\{|h_{ab}{|}^{2}\geq \frac{\gamma_{th}\left(P_{j}{\left|h_{jb}\right|}^{2}+2\sigma_{b}^{2}\right)}{2P_{\max}}\right\}.$$

When Alice transmits covert messages, the signal received at Bob can be given by

(5) $$y_{b}\left[i\right]=\sqrt{P_{r,1}}h_{ab}x_{p}\left[i\right]+\sqrt{P_{c}}h_{ab}x_{c}\left[i\right]+\delta \sqrt{P_{j}}h_{jb}x_{j}\left[i\right]+n_{b}\left[i\right],$$

where $P_{r,1}$ denotes the transmit power of Alice for public messages, $P_{c}$ denotes the transmit power of Alice for covert messages, and $x_{c}\left[i\right]$ denotes the transmit signal for covert messages. The received SINR at Bob can be given by

(6) $$\begin{array}{ccc} {\mathrm{SIN}R}_{b} & = & \frac{P_{r,1}{\left|h_{ab}\right|}^{2}}{\delta T_{S}/TP_{j}{\left|h_{jb}\right|}^{2}+P_{c}{\left|h_{ab}\right|}^{2}+\sigma_{b}^{2}}\\ & = & \frac{2P_{r,1}{\left|h_{ab}\right|}^{2}}{\delta P_{j}{\left|h_{jb}\right|}^{2}+2P_{c}{\left|h_{ab}\right|}^{2}+2\sigma_{b}^{2}}. \end{array}$$

To ensure that Bob can decode the public messages successfully, $P_{r,1}$ can be given by

(7) $$P_{r,1}=\gamma_{th}P_{c}+\frac{\gamma_{th}\left(P_{j}{\left|h_{jb}\right|}^{2}+2\sigma_{b}^{2}\right)}{2{\left|h_{ab}\right|}^{2}}.$$

Considering the maximum power constraint at Alice, i.e., $P_{r,1}+P_{c}\leq P_{\max}$, the necessary condition for Alice to perform its covert transmission can be given by

(8) $$B=\left\{|h_{ab}{|}^{2}\geq \frac{\gamma_{th}\left(P_{j}{\left|h_{jb}\right|}^{2}+2\sigma_{b}^{2}\right)}{2P_{\max}-2\left(1+\gamma_{th}\right)P_{c}}\right\}.$$

### 2.2. Detection Performance of CJ

In the first phase, the CJ detects whether Alice transmits its covert messages. The received signal at the CJ can be given by

(9) $$y_{j}\left[i\right]=\left\{\begin{array}{cc} \sqrt{P_{r,0}}h_{aj}x_{p}\left[i\right]+n_{j}\left[i\right], & H_{0}\\ \sqrt{P_{r,1}}h_{aj}x_{p}\left[i\right]+\sqrt{P_{c}}h_{aj}x_{c}\left[i\right]+n_{j}\left[i\right], & H_{1} \end{array}\right.,$$

where $n_{j}\left[i\right]$∼$\mathrm{CN}(0,\sigma_{j}^{2})$ denotes the AWGN at CJ; and $H_{1}$ and $H_{0}$ denote the hypotheses that Alice transmits with or without the covert message, respectively. The optimal detection scheme can be given by [20]

(10) $$\begin{array}{ccc} T_{CJ} & = & \frac{1}{T_{J}/Tn}\sum_{i=1}^{T_{J}/Tn}{\left|y_{j}\left[i\right]\right|}^{2}\overset{D_{j,1}}{\underset{D_{j,0}}{≷}}\tau_{j}\\ & = & \frac{1}{1/2n}\sum_{i=1}^{1/2n}{\left|y_{j}\left[i\right]\right|}^{2}\overset{D_{j,1}}{\underset{D_{j,0}}{≷}}\tau_{j}, \end{array}$$

where $\tau_{j}$ is the detection threshold at CJ; and $D_{j,1}$ and $D_{j,0}$ are the binary decisions, corresponding to Alice transmitting with or without the covert message, respectively. Assuming *n* is large enough and applying the strong law of large numbers, we can have

(11) $$T_{CJ}=\left\{\begin{array}{cc} P_{r,0}{\left|h_{aj}\right|}^{2}+\sigma_{j}^{2}, & H_{0}\\ P_{r,1}{\left|h_{aj}\right|}^{2}+P_{c}{\left|h_{aj}\right|}^{2}+\sigma_{j}^{2}, & H_{1} \end{array}\right..$$

Thus, the probability of false alarm (FA) $\alpha_{j}$ and the probability of missed detection (MD) $\beta_{j}$ for the CJ can be given by

(12) $$\begin{array}{cc} \alpha_{j} & =\mathrm{Pr}\left\{P_{r,0}{\left|h_{aj}\right|}^{2}+\sigma_{j}^{2}>\tau_{j}\;|\;A\right\}\\ \; & =\mathrm{Pr}\left\{\frac{\gamma_{\mathrm{th}}\left(P_{j}{\left|h_{jb}\right|}^{2}+2\sigma_{b}^{2}\right)}{2{\left|h_{ab}\right|}^{2}}{\left|h_{aj}\right|}^{2}+\sigma_{j}^{2}>\tau_{j}\;|\;A\right\}\\ \; & =\left\{\begin{array}{cc} 1, & \tau_{j}<\sigma_{j}^{2}\\ \frac{\mathrm{Pr}\left(B\leq |h_{ab}{|}^{2}<A\right)}{\mathrm{Pr}\left(|h_{ab}{|}^{2}\geq B\right)}, & \sigma_{j}^{2}\leq \tau_{j}<a_{1}\\ 0, & \tau_{j}\geq a_{1} \end{array}\right.\\ \; & =\left\{\begin{array}{cc} 1, & \tau_{j}<\sigma_{j}^{2}\\ p_{\mathrm{FA},j}\left(\tau_{j}\right), & \sigma_{j}^{2}\leq \tau_{j}<a_{1}\\ 0, & \tau_{j}\geq a_{1} \end{array}\right., \end{array}$$

where $a_{1}=P_{\max}{\left|h_{aj}\right|}^{2}+\sigma_{j}^{2}$, $A=\frac{\gamma_{\mathrm{th}}(P_{j}|h_{jb}{|}^{2}+2\sigma_{b}^{2})|h_{aj}{|}^{2}}{2(\tau_{j}-\sigma_{j}^{2})}$, $B=\frac{\gamma_{\mathrm{th}}(P_{j}{|h_{jb}|}^{2}+2\sigma_{b}^{2})}{2P_{\max}}$, $p_{FA,j}\left(\tau_{j}\right)=1-\exp\left(\bar{\omega }\left(\frac{1}{P_{\max}}-\frac{|h_{aj}{|}^{2}}{\tau_{j}-\sigma_{j}^{2}}\right)\right)$ and $\bar{\omega }=\gamma_{th}(1/2P_{j}|h_{jb}{|}^{2}+\sigma_{b}^{2})/\lambda_{ab}$.

(13) $$\begin{array}{cc} \beta_{j} & =\mathrm{Pr}\left\{P_{r,1}{\left|h_{aj}\right|}^{2}+P_{c}{\left|h_{aj}\right|}^{2}+\sigma_{j}^{2}<\tau_{j}\;|\;B\right\}\\ \; & =\mathrm{Pr}\{\left(\gamma_{\mathrm{th}}P_{c}+\frac{\gamma_{\mathrm{th}}\left(P_{j}{\left|h_{jb}\right|}^{2}+2\sigma_{b}^{2}\right)}{2{\left|h_{ab}\right|}^{2}}\right){\left|h_{aj}\right|}^{2}\\ \; & \;+P_{c}{\left|h_{aj}\right|}^{2}+\sigma_{j}^{2}<\tau_{j}\;|\;B\}\\ \; & =\left\{\begin{array}{cc} 0, & \tau_{j}<a_{2}\\ p_{\mathrm{MD},j}\left(\tau_{j}\right), & a_{2}\leq \tau_{j}<a_{1}\\ 1, & \tau_{j}\geq a_{1} \end{array}\right., \end{array}$$

where $a_{2}=\left(1+\gamma_{th}\right)P_{c}{\left|h_{aj}\right|}^{2}+\sigma_{j}^{2}$, $a_{1}\geq a_{2}$ and $p_{MD,j}\left(\tau_{j}\right)=\exp\left(\bar{\omega }\left(\frac{1}{P_{\max}-\left(1+\gamma_{th}\right)P_{c}}-\frac{|h_{aj}{|}^{2}}{\tau_{j}-\left(1+\gamma_{th}\right)P_{c}{\left|h_{aj}\right|}^{2}-\sigma_{j}^{2}}\right)\right)$. We assume that the probability of Alice transmitting covert messages is ρ. Without loss of generality, we set ρ=1/2. The DEP of CJ is expressed as

(14) $$\xi_{j}=\frac{1}{2}\left(\alpha_{j}+\beta_{j}\right).$$

With Equations (12)–(14), the DEP of the CJ can be given by

(15) $$\xi_{j}=\left\{\begin{array}{cc} \frac{1}{2}, & \tau_{j}<\sigma_{j}^{2}\\ \frac{1}{2}p_{FA,j}\left(\tau_{j}\right), & \sigma_{j}^{2}\leq \tau_{j}<a_{2}\\ \frac{1}{2}\left[p_{FA,j}\left(\tau_{j}\right)+p_{MD,j}\left(\tau_{j}\right)\right], & a_{2}\leq \tau_{j}<a_{1}\\ \frac{1}{2}, & \tau_{j}\geq a_{1} \end{array}\right..$$

As per (15), we differentiate $p_{FA,j}\left(\tau_{j}\right)$ with respect to $\tau_{j}$ to obtain

(16) $$\frac{\partial p_{FA,j}\left(\tau_{j}\right)}{\partial \tau_{j}}=-\frac{\bar{\omega }{\left|h_{aj}\right|}^{2}}{{\left(\tau_{j}-\sigma_{j}^{2}\right)}^{2}}\exp\left(\bar{\omega }\left(\frac{1}{P_{\max}}-\frac{|h_{aj}{|}^{2}}{\tau_{j}-\sigma_{j}^{2}}\right)\right).$$

Obviously, it is observed that $\xi_{j}$ is a monotonically decreasing function of $\tau_{j}$ over the interval $\sigma_{j}^{2}\leq \tau_{j}<a_{2}$. We can easily observe that $\xi_{j}=1/2$ in the intervals $\tau_{j}<\sigma_{j}^{2}$ and $\tau_{j}\geq a_{1}$. Hence, there exists an optimal $\tau_{j}$ that minimizes $\xi_{j}$, which falls into the interval $a_{2}\leq \tau_{j}<a_{1}$, i.e.,

(17) $$\tau_{j}^{opt}=\underset{a_{2}\leq \tau_{j}<a_{1}}{\arg\min}\frac{1}{2}\left[p_{FA,j}\left(\tau_{j}\right)+p_{MD,j}\left(\tau_{j}\right)\right].$$

Equation (17) can be solved through a numerical search. By substituting (17) into (14), the minimum DEP of CJ can be obtained, i.e., $\xi_{j}^{opt}=1/2\left[\alpha_{j}\left(\tau_{j}^{opt}\right)+\beta_{j}\left(\tau_{j}^{opt}\right)\right]$.

### 2.3. Detection Performance of Willie

#### 2.3.1. The Detection Threshold of Willie

We consider the worst-case scenario for Alice’s covert communication, i.e., Willie can achieve the best detection performance. Therefore, for deriving Willie’s optimal detection threshold, it is assumed that Willie knows the CJ’s behavior. Therefore, the received signal at Willie can be given by

(18) $$y_{w}\left[i\right]=\left\{\begin{array}{cc} \sqrt{P_{r,0}}h_{aw}x_{p}\left[i\right]+\sqrt{P_{j}}h_{jw}x_{j}\left[i\right]+n_{w}\left[i\right], & H_{0}\\ \sqrt{P_{r,1}}h_{aw}x_{p}\left[i\right]+\sqrt{P_{c}}h_{aw}x_{c}\left[i\right]+n_{w}\left[i\right], & H_{1} \end{array}\right.,$$

where $n_{w}\left[i\right]$∼$\mathrm{CN}(0,\sigma_{w}^{2})$ denotes the AWGN at Willie. The probability of FA $\alpha_{w,1}$ and the probability of MD $\beta_{w,1}$ can be given by

(19) $$\begin{array}{ccc} \alpha_{w,1} & = & \mathrm{Pr}\left\{P_{r,0}{\left|h_{aw}\right|}^{2}+1/2P_{j}{\left|h_{jw}\right|}^{2}+\sigma_{w}^{2}>\tau_{w}|\;A\right\}\\ & = & \left\{\begin{array}{cc} 1, & \tau_{w}<b_{2}\\ p_{FA,w}\left(\tau_{w}\right), & b_{2}\leq \tau_{w}<b_{1}\\ 0, & \tau_{w}\geq b_{1} \end{array}\right., \end{array}$$

where $b_{1}=P_{\max}|h_{aw}{|}^{2}+1/2P_{j}{\left|h_{jw}\right|}^{2}+\sigma_{w}^{2}$, $b_{2}=1/2P_{j}{\left|h_{jw}\right|}^{2}+\sigma_{w}^{2}$ and

(20) $$\begin{array}{cc} p_{FA,w}\left(\tau_{w}\right)= & \;\frac{\mathrm{Pr}\left\{\frac{\gamma_{th}\left(P_{j}{\left|h_{jb}\right|}^{2}+2\sigma_{b}^{2}\right)}{2P_{\max}}\leq {\left|h_{ab}\right|}^{2}<\phi \right\}}{\mathrm{Pr}\left\{|h_{ab}{|}^{2}\geq \frac{\gamma_{th}\left(P_{j}{\left|h_{jb}\right|}^{2}+2\sigma_{b}^{2}\right)}{2P_{\max}}\right\}}\\ = & \;1-\frac{\left(P_{\max}\lambda_{ab}+b_{3}\right)\left(\tau_{w}-b_{2}\right)}{P_{\max}\left(\left(\tau_{w}-b_{2}\right)\lambda_{ab}+b_{3}{\left|h_{aw}\right|}^{2}\right)}\exp\left\{\frac{\gamma_{th}\sigma_{b}^{2}}{P_{\max}\lambda_{ab}}-\frac{\gamma_{th}\sigma_{b}^{2}{\left|h_{aw}\right|}^{2}}{\left(\tau_{w}-b_{2}\right)\lambda_{ab}}\right\}, \end{array}$$

where $b_{3}=1/2\gamma_{th}P_{j}\lambda_{jb}$ and $\phi =\frac{\gamma_{th}\left(1/2P_{j}{\left|h_{jb}\right|}^{2}+\sigma_{b}^{2}\right){\left|h_{aw}\right|}^{2}}{\tau_{w}-1/2P_{j}{\left|h_{jw}\right|}^{2}-\sigma_{w}^{2}}$.

(21) $$\begin{array}{ccc} \beta_{w,1} & = & \mathrm{Pr}\left\{P_{r,1}{\left|h_{aw}\right|}^{2}+P_{c}{\left|h_{aw}\right|}^{2}+\sigma_{w}^{2}<\tau_{w}|\;B\right\}\\ & = & \left\{\begin{array}{cc} 0, & \tau_{w}<c_{2}\\ p_{MD,w}\left(\tau_{w}\right), & c_{2}\leq \tau_{w}<c_{1}\\ 1, & \tau_{w}\geq c_{1} \end{array}\right., \end{array}$$

where $c_{1}=P_{\max}{\left|h_{aw}\right|}^{2}+\sigma_{w}^{2}$, $c_{2}=\left(1+\gamma_{th}\right)P_{c}{\left|h_{aw}\right|}^{2}+\sigma_{w}^{2}$ and

(22) $$\begin{array}{cc} p_{MD,w}\left(\tau_{w}\right) & =\frac{\left(\zeta \lambda_{ab}+b_{3}\right)\left(\tau_{w}-c_{2}\right)}{\zeta \left(\left(\tau_{w}-c_{2}\right)\lambda_{ab}+b_{3}{\left|h_{aw}\right|}^{2}\right)}\exp\left\{\frac{\gamma_{th}\sigma_{b}^{2}}{\zeta \lambda_{ab}}-\frac{\gamma_{th}\sigma_{b}^{2}{\left|h_{aw}\right|}^{2}}{\left(\tau_{w}-c_{2}\right)\lambda_{ab}}\right\}, \end{array}$$

where $\zeta =P_{\max}-\left(1+\gamma_{th}\right)P_{c}$. With (14), (19) and (21), the DEP of Willie under three cases can be given as follows.

Case 1: $b_{2}<c_{2}$
  (23) $$\xi_{w,1}=\left\{\begin{array}{cc} \frac{1}{2}, & \tau_{w}<b_{2}\\ \frac{1}{2}p_{FA,w}\left(\tau_{w}\right), & b_{2}\leq \tau_{w}<c_{2}\\ \frac{1}{2}\left[p_{FA,w}\left(\tau_{w}\right)+p_{MD,w}\left(\tau_{w}\right)\right], & c_{2}\leq \tau_{w}<c_{1}\\ \frac{1}{2}+\frac{1}{2}p_{FA,w}\left(\tau_{w}\right), & c_{1}\leq \tau_{w}<b_{1}\\ \frac{1}{2}, & \tau_{w}\geq b_{1} \end{array}\right..$$

As per (20), we differentiate $p_{FA,w}\left(\tau_{w}\right)$ with respect to $\tau_{w}$ to obtain

(24) $$\begin{array}{cc} \frac{\partial p_{\mathrm{FA},w}\left(\tau_{w}\right)}{\partial \tau_{w}}\; & =-\left[\frac{\kappa }{\left(\tau_{w}-b_{2}\right)\lambda_{ab}+\kappa }+\frac{\gamma_{\mathrm{th}}\sigma_{b}^{2}{\left|h_{aw}\right|}^{2}}{\left(\tau_{w}-b_{2}\right)\lambda_{ab}}\right]\frac{P_{\max}\lambda_{ab}+b_{3}}{P_{\max}\left(\left(\tau_{w}-b_{2}\right)\lambda_{ab}+\kappa \right)}\times \\ \; & \;\exp\left\{\frac{\gamma_{\mathrm{th}}\sigma_{b}^{2}}{P_{\max}\lambda_{ab}}-\frac{\gamma_{\mathrm{th}}\sigma_{b}^{2}{\left|h_{aw}\right|}^{2}}{\left(\tau_{w}-b_{2}\right)\lambda_{ab}}\right\}, \end{array}$$

where $\kappa =1/2\gamma_{th}P_{j}\lambda_{jb}{\left|h_{aw}\right|}^{2}$. It is easy to see that within the intervals $b_{2}\leq \tau_{w}<c_{2}$ and $c_{1}\leq \tau_{w}<b_{1}$, $\partial p_{FA,w}\left(\tau_{w}\right)/\partial \tau_{w}<0$; hence, $\xi_{w,1}$ is a decreasing function with respect to $\tau_{w}$. We also observe that $\xi_{w,1}=1/2$ when $\tau_{w}<b_{2}$ or $\tau_{w}\geq b_{1}$. Given that $p_{FA,w}\left(\tau_{w}\right)\geq 0$, it follows that $\xi_{w,1}\geq 1/2$ over $c_{1}\leq \tau_{w}<b_{1}$. Since $\xi_{w,1}$ is a continuous function, the optimal value of $\tau_{w}$ minimizing $\xi_{w,1}$ falls into the interval $c_{2}\leq \tau_{w}<c_{1}$, i.e.,

(25) $$\tau_{w}^{opt}=\underset{c_{2}\leq \tau_{w}<c_{1}}{\arg\min}\frac{1}{2}\left[p_{FA,w}\left(\tau_{w}\right)+p_{MD,w}\left(\tau_{w}\right)\right].$$

Based on the preceding analysis and the substitution of (25) into (23), it can be concluded that the minimum DEP of Willie satisfies $\xi_{w,1}\left(\tau_{w}^{opt}\right)<1/2$.

Case 2: $c_{2}\leq b_{2}<c_{1}$
  (26) $$\xi_{w,1}=\left\{\begin{array}{cc} \frac{1}{2}, & \tau_{w}<c_{2}\\ \frac{1}{2}+\frac{1}{2}p_{MD,w}\left(\tau_{w}\right), & c_{2}\leq \tau_{w}<b_{2}\\ \frac{1}{2}\left[p_{FA,w}\left(\tau_{w}\right)+p_{MD,w}\left(\tau_{w}\right)\right], & b_{2}\leq \tau_{w}<c_{1}\\ \frac{1}{2}+\frac{1}{2}p_{FA,w}\left(\tau_{w}\right), & c_{1}\leq \tau_{w}<b_{1}\\ \frac{1}{2}, & \tau_{w}\geq b_{1} \end{array}\right..$$

As per (22), we differentiate $p_{MD,w}\left(\tau_{w}\right)$ with respect to $\tau_{w}$ to obtain

(27) $$\begin{array}{cc} \frac{\partial p_{MD,w}\left(\tau_{w}\right)}{\partial \tau_{w}} & =\left[\frac{\kappa }{\left(\tau_{w}-c_{2}\right)\lambda_{ab}+\kappa }+\frac{\gamma_{th}\sigma_{b}^{2}{\left|h_{aw}\right|}^{2}}{\left(\tau_{w}-c_{2}\right)\lambda_{ab}}\right]\\ \; & \;\times \frac{\zeta \lambda_{ab}+b_{3}}{\zeta \left(\left(\tau_{w}-c_{2}\right)\lambda_{ab}+\kappa \right)}\\ \; & \;\times \exp\left\{\frac{\gamma_{th}\sigma_{b}^{2}}{\zeta \lambda_{ab}}-\frac{\gamma_{th}\sigma_{b}^{2}{\left|h_{aw}\right|}^{2}}{\left(\tau_{w}-c_{2}\right)\lambda_{ab}}\right\}. \end{array}$$

We can observe that $\partial p_{MD,w}\left(\tau_{w}\right)/\partial \tau_{w}>0$ both in the interval $c_{2}\leq \tau_{w}<b_{2}$ and in $c_{1}\leq \tau_{w}<b_{1}$; hence, $\xi_{w,1}$ is an increasing function with respect to $\tau_{w}$. In the intervals $c_{2}\leq \tau_{w}<b_{2}$ and $c_{1}\leq \tau_{w}<b_{1}$, we have $\xi_{w,1}\geq 1/2$, while $\xi_{w,1}=1/2$ for $\tau_{w}<c_{2}$ and $\tau_{w}\geq b_{1}$. It can be inferred that within the intervals discussed, the minimum value of $\xi_{w,1}$ is 1/2. Therefore, to find the minimum $\xi_{w,1}$, we need to examine its value in the remaining interval $b_{2}\leq \tau_{w}<c_{1}$. We define it in this interval as

(28) $$\xi_{w,1}=\frac{1}{2}\left(1-\frac{f(\tau_{w}+c_{2}-b_{2})}{f(b_{1}-b_{2}+c_{2})}+\frac{f(\tau_{w})}{f(c_{1})}\right),$$

where $f\left(x\right)=\frac{\left(x-c_{2}\right)\lambda_{ab}}{\left(x-c_{2}\right)\lambda_{ab}+b_{3}{\left|h_{aw}\right|}^{2}}e^{-\frac{\gamma_{th}\sigma_{b}^{2}{\left|h_{aw}\right|}^{2}}{\left(x-c_{2}\right)\lambda_{ab}}}$. From (27), we have $f^{\prime }\left(x\right)>0$; hence, f(x) is an increasing function of *x*. Since $b_{1}-b_{2}>c_{1}-c_{2}$ and $b_{2}\geq c_{2}$, we have $f\left(b_{1}-b_{2}+c_{2}\right)>f\left(c_{1}\right)$ and $f\left(\tau_{w}\right)>f\left(\tau_{w}+c_{2}-b_{2}\right)$, thus implying $f\left(\tau_{w}\right)/f\left(c_{1}\right)>f\left(\tau_{w}+c_{2}-b_{2}\right)/f\left(b_{1}-b_{2}+c_{2}\right)$. Consequently, for $b_{2}\leq \tau_{w}<c_{1}$, $\xi_{w,1}>1/2$ always holds. In the interval $c_{2}\leq b_{2}<c_{1}$, Willie’s minimum DEP is 1/2, and the optimal value of $\tau_{w}$ can be expressed as $c_{2}$.

Case 3: $b_{2}\geq c_{1}$
  (29) $$\xi_{w,1}=\left\{\begin{array}{cc} \frac{1}{2}, & \tau_{w}<c_{2}\\ \frac{1}{2}+\frac{1}{2}p_{MD,w}\left(\tau_{w}\right), & c_{2}\leq \tau_{w}<c_{1}\\ 1, & c_{1}\leq \tau_{w}<b_{2}\\ \frac{1}{2}+\frac{1}{2}p_{FA,w}\left(\tau_{w}\right), & b_{2}\leq \tau_{w}<b_{1}\\ \frac{1}{2}, & \tau_{w}\geq b_{1} \end{array}\right..$$

Here, $\xi_{w,1}$ is a continuous function. It is readily observed that $\xi_{w,1}\geq 1/2$ for $c_{2}\leq \tau_{w}<c_{1}$ and $b_{2}\leq \tau_{w}<b_{1}$. Hence, Willie’s minimum DEP is 1/2, and the optimal value of $\tau_{w}$ can be express as $c_{2}$.

We consider the case $c_{2}\geq b_{2}$, which minimizes the DEP of Willie. Consequently, Willie’s optimal detection threshold can be given by (25).

#### 2.3.2. The DEP of Willie

In practice, the received signal at Willie can be given by

(30) $$y_{w}\left[i\right]=\left\{\begin{array}{cc} \begin{array}{c} \sqrt{P_{r,0}}h_{aw}x_{p}\left[i\right]+\delta \sqrt{P_{j}}h_{jw}x_{j}\left[i\right]+n_{w}\left[i\right], \end{array} & H_{0}\\ \begin{array}{c} \sqrt{P_{r,1}}h_{aw}x_{p}\left[i\right]+\sqrt{P_{c}}h_{aw}x_{c}\left[i\right]+\delta \sqrt{P_{j}}h_{jw}x_{j}\left[i\right]+n_{w}\left[i\right], \end{array} & H_{1} \end{array}\right..$$

The probability of FA $\alpha_{w}$ and the probability of MD $\beta_{w}$ in practice can be given by

(31) $$\begin{array}{cc} \alpha_{w} & =\mathrm{Pr}\left\{P_{r,0}{\left|h_{aw}\right|}^{2}+1/2\delta P_{j}{\left|h_{jw}\right|}^{2}+\sigma_{w}^{2}>\tau_{w}|\;A\right\}\\ \; & =\mathrm{Pr}\left\{D_{j,1}|\;H_{0}\right\}\mathrm{Pr}\left\{P_{r,0}{\left|h_{aw}\right|}^{2}+\sigma_{w}^{2}>\tau_{w}|\;A\right\}+\mathrm{Pr}\left\{D_{j,0}|\;H_{0}\right\}\times \\ \; & \;\mathrm{Pr}\left\{P_{r,0}{\left|h_{aw}\right|}^{2}+1/2P_{j}{\left|h_{jw}\right|}^{2}+\sigma_{w}^{2}>\tau_{w}|\;A\right\}\\ \; & =\alpha_{j}^{*}\mathrm{Pr}\left\{P_{r,0}{\left|h_{aw}\right|}^{2}+\sigma_{w}^{2}>\tau_{w}|\;A\right\}+\left(1-\alpha_{j}^{*}\right)\times \\ \; & \;\mathrm{Pr}\left\{P_{r,0}{\left|h_{aw}\right|}^{2}+1/2P_{j}{\left|h_{jw}\right|}^{2}+\sigma_{w}^{2}>\tau_{w}|\;A\right\}\\ \; & =\alpha_{j}^{*}\alpha_{w,2}\left(\tau_{w}\right)+\left(1-\alpha_{j}^{*}\right)\alpha_{w,1}\left(\tau_{w}\right), \end{array}$$

where $\alpha_{j}^{*}=\alpha_{j}\left(\tau_{j}^{opt}\right)$ and

(32) $$\alpha_{w,2}=\left\{\begin{array}{cc} 1, & \tau_{w}<\sigma_{w}^{2}\\ p_{FA,w}\left(\tau_{w}+1/2P_{j}{\left|h_{jw}\right|}^{2}\right), & \sigma_{w}^{2}\leq \tau_{w}<c_{1}\\ 0, & \tau_{w}\geq c_{1} \end{array}\right..$$

(33) $$\begin{array}{cc} \beta_{w} & =\mathrm{Pr}\left\{P_{r,1}{\left|h_{aw}\right|}^{2}+P_{c}{\left|h_{aw}\right|}^{2}+1/2\delta P_{j}{\left|h_{jw}\right|}^{2}+\sigma_{w}^{2}<\tau_{w}|\;B\right\}\\ \; & =\mathrm{Pr}\left\{D_{j,0}|\;H_{1}\right\}\mathrm{Pr}\left\{P_{r,1}{\left|h_{aw}\right|}^{2}+P_{c}{\left|h_{aw}\right|}^{2}+1/2P_{j}{\left|h_{jw}\right|}^{2}+\sigma_{w}^{2}<\tau_{w}|\;B\right\}+\\ \; & \;\mathrm{Pr}\left\{D_{j,1}|\;H_{1}\right\}\times \mathrm{Pr}\left\{P_{r,1}{\left|h_{aw}\right|}^{2}+P_{c}{\left|h_{aw}\right|}^{2}+\sigma_{w}^{2}<\tau_{w}|\;B\right\}\\ \; & =\beta_{j}^{*}\mathrm{Pr}\left\{P_{r,1}{\left|h_{aw}\right|}^{2}+P_{c}{\left|h_{aw}\right|}^{2}+1/2P_{j}{\left|h_{jw}\right|}^{2}+\sigma_{w}^{2}<\tau_{w}|\;B\right\}+\left(1-\beta_{j}^{*}\right)\times \\ \; & \;\mathrm{Pr}\left\{P_{r,1}{\left|h_{aw}\right|}^{2}+P_{c}{\left|h_{aw}\right|}^{2}+\sigma_{w}^{2}<\tau_{w}|\;B\right\}\\ \; & =\beta_{j}^{*}\beta_{w,2}\left(\tau_{w}\right)+\left(1-\beta_{j}^{*}\right)\beta_{w,1}\left(\tau_{w}\right), \end{array}$$

where $\beta_{j}^{*}=\beta_{j}\left(\tau_{j}^{opt}\right)$ and

(34) $$\beta_{w,2}=\left\{\begin{array}{cc} 0, & \tau_{w}<c_{3}\\ p_{MD,w}\left(\tau_{w}-1/2P_{j}{\left|h_{jw}\right|}^{2}\right), & c_{3}\leq \tau_{w}<b_{1}\\ 1, & \tau_{w}\geq b_{1} \end{array}\right.,$$

where $c_{3}=\left(1+\gamma_{th}\right)P_{c}|h_{aw}{|}^{2}+1/2P_{j}{\left|h_{jw}\right|}^{2}+\sigma_{w}^{2}$. Finally, by combining (14), (25), (31) and (33), the DEP of Willie in practice can be given by

(35) $$\begin{array}{ccc} \xi_{w,2} & = & \frac{1}{2}\left[\left(1-\alpha_{j}^{*}\right)\alpha_{w,1}\left(\tau_{w}^{opt}\right)+\alpha_{j}^{*}\alpha_{w,2}\left(\tau_{w}^{opt}\right)+\left(1-\beta_{j}^{*}\right)\beta_{w,1}\left(\tau_{w}^{opt}\right)+\beta_{j}^{*}\beta_{w,2}\left(\tau_{w}^{opt}\right)\right]. \end{array}$$

### 2.4. Average Covert Rate

The ACR achieved by the considered system is derived in the following theorem.

**Theorem 1.** 
*Given fixed transmit power $P_{c}$ and jamming power $P_{j}$, the average covert rate achieved by the considered system can be given by*

(36) $$\begin{array}{cc} \;R_{c,avg} & =\frac{1}{\ln2}e^{-\frac{b}{\lambda_{ab}}}\ln\left(1+\frac{P_{c}b}{\sigma_{b}^{2}}\right)+\frac{e^{\frac{\sigma_{b}^{2}}{P_{c}\lambda_{ab}}}}{\ln2}E_{1}\left(c\right)-\frac{a\lambda_{jb}e^{-\frac{b}{a\lambda_{jb}}}}{\left(a\lambda_{jb}+\lambda_{ab}\right)\ln2}e^{-\left(\frac{1}{\lambda_{ab}}+\frac{1}{a\lambda_{jb}}\right)b}\times \\ \; & \;\ln\left(1+\frac{P_{c}b}{\sigma_{b}^{2}}\right)-\frac{a\lambda_{jb}e^{-\frac{b}{a\lambda_{jb}}}}{\left(a\lambda_{jb}+\lambda_{ab}\right)\ln2}e^{\left(\frac{1}{\lambda_{ab}}+\frac{1}{a\lambda_{jb}}\right)\frac{\sigma_{b}^{2}}{P_{c}}}E_{1}\left(d\right)+\frac{1}{2}\beta_{j}^{*}\times \\ \; & \;E\left[\log_{2}\left(1+\frac{P_{c}{\left|h_{ab}\right|}^{2}}{1/2P_{j}{\left|h_{jb}\right|}^{2}+\sigma_{b}^{2}}\right),B\right], \end{array}$$

*where $a=\frac{1/2\gamma_{th}P_{j}}{P_{\max}-\left(1+\gamma_{th}\right)P_{c}}$, $b=\frac{\gamma_{th}\sigma_{b}^{2}}{P_{\max}-\left(1+\gamma_{th}\right)P_{c}}$, $c=\frac{P_{c}b+\sigma_{b}^{2}}{P_{c}\lambda_{ab}}$, $d=\frac{\left(P_{c}b+\sigma_{b}^{2}\right)\left(a\lambda_{jb}+\lambda_{ab}\right)}{P_{c}a\lambda_{ab}\lambda_{jb}}$, and $E_{1}\left(x\right)=\int_{x}^{\infty }e^{-t}/tdt$ denotes the exponential integral function.*

**Proof of Theorem 1.**  After decoding Alice’s public messages, Bob removes the public component from his received signal. Therefore, the SINR for decoding covert messages can be given by:

(37) $${\mathrm{SIN}R}_{c}=\frac{P_{c}{\left|h_{ab}\right|}^{2}}{1/2\delta P_{j}{\left|h_{jb}\right|}^{2}+\sigma_{b}^{2}}.$$

The covert rate is defined as $R_{c}=\log_{2}\left(1+{\mathrm{SIN}R}_{c}\right)$, and the average covert rate can be given by

(38) $$\begin{array}{cc} R_{c,avg} & =\rho E\left[\log_{2}\left(1+{\mathrm{SIN}R}_{c}\right),B\right]\\ \; & =\frac{1}{2}\mathrm{Pr}\{D_{j,1}|\;H_{1}\}E\left[\log_{2}\left(1+\frac{P_{c}{\left|h_{ab}\right|}^{2}}{\sigma_{b}^{2}}\right),B\right]+\frac{1}{2}\mathrm{Pr}\{D_{j,0}|\;H_{1}\}\times \\ \; & \;E\left[\log_{2}\left(1+\frac{P_{c}{\left|h_{ab}\right|}^{2}}{1/2P_{j}{\left|h_{jb}\right|}^{2}+\sigma_{b}^{2}}\right),B\right]\\ \; & =\frac{1}{2}\left(1-\beta_{j}^{*}\right)E\left[\log_{2}\left(1+\frac{P_{c}{\left|h_{ab}\right|}^{2}}{\sigma_{b}^{2}}\right),B\right]+\frac{1}{2}\beta_{j}^{*}E\left[\log_{2}\left(1+\frac{P_{c}{\left|h_{ab}\right|}^{2}}{1/2P_{j}{\left|h_{jb}\right|}^{2}+\sigma_{b}^{2}}\right),B\right]\\ \; & =\frac{1}{2}\left(1-\beta_{j}^{*}\right)\int_{b}^{+\infty }\int_{0}^{\frac{y-b}{a}}\log_{2}\left(1+\frac{P_{c}y}{\sigma_{b}^{2}}\right)f_{|h_{ab}{|}^{2}}\left(y\right)f_{|h_{jb}{|}^{2}}\left(x\right)\;dx\;dy+\frac{1}{2}\beta_{j}^{*}\int_{0}^{+\infty }\int_{ax+b}^{+\infty }\\ \; & \;\log_{2}\left(1+\frac{P_{c}y}{1/2P_{j}x+\sigma_{b}^{2}}\right)f_{|h_{ab}{|}^{2}}\left(y\right)f_{|h_{jb}{|}^{2}}\left(x\right)dy\;dx\\ \; & =\frac{1}{2\ln2}\left(1-\beta_{j}^{*}\right)\int_{b}^{+\infty }\frac{1}{\lambda_{ab}}e^{-\frac{y}{\lambda_{ab}}}\left(1-e^{-\frac{y-b}{a\lambda_{ab}}}\right)\ln\left(1+\frac{P_{c}y}{\sigma_{b}^{2}}\right)dy\;+\frac{1}{2}\beta_{j}^{*}\int_{0}^{+\infty }\int_{ax+b}^{+\infty }\\ \; & \;\log_{2}\left(1+\frac{P_{c}y}{1/2P_{j}x+\sigma_{b}^{2}}\right)f_{|h_{ab}{|}^{2}}\left(y\right)f_{|h_{jb}{|}^{2}}\left(x\right)dy\;dx, \end{array}$$

where $f_{|h_{ab}{|}^{2}}\left(y\right)=\frac{1}{\lambda_{ab}}e^{-\frac{y}{\lambda_{ab}}}$ and $f_{|h_{jb}{|}^{2}}\left(x\right)=\frac{1}{\lambda_{jb}}e^{-\frac{x}{\lambda_{jb}}}$. By employing the integral solution from [32] (Equation (4.337.1)) to solve the first integral, we obtain the expression for the first term. Combining this result with the second integral gives (36). □

## 3. Results

In this section, numerical results are provided to evaluate the covert performance. All simulations in this study were performed using Matlab. Without loss of generality, we assume that $\lambda_{ab}=\lambda_{jb}=1$ and $P_{\max}=10\;W$.

**Figure 2:**
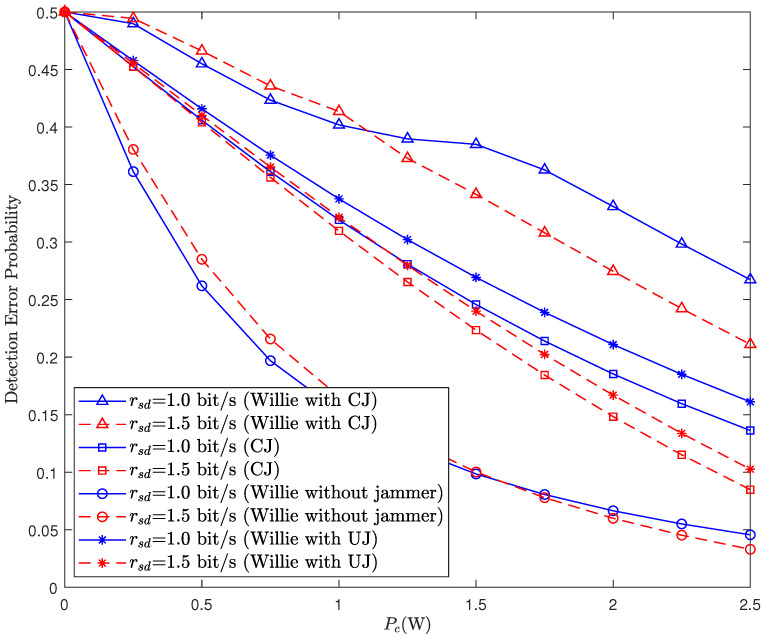
Detection error probability versus covert transmit power $P_{c}$ for different $r_{sd}$ and $P_{j}$.

Figure 2 illustrates the relationship between the detection error probability and the covert transmit power for Willie and the jammer. The covert constraint in the considered system is given by $\xi_{w,2}\geq min\left\{\rho ,1-\rho \right\}-\varepsilon$, with ε≥0 [33]. There is a contradiction regarding the impact of $P_{c}$ on covert communication. On the one hand, it is obvious that an increase in $P_{c}$ can increase the risk of the exposure of covert transmission. For instance, the DEPs of Willie for the scheme with an uninformed jammer (UJ) and the scheme without a jammer decrease monotonically as $P_{c}$ increases. On the other hand, we can see that the DEP of CJ also decreases monotonically with increasing $P_{c}$, indicating that increasing $P_{c}$ can enhance the detection accuracy of the cognitive jammer, which is beneficial for facilitating covert communication. However, between the two conflicting parties, the first party holds the dominant position. Thus, it can be observed that the DEP of Willie for the scheme with CJ decreases monotonically with increasing $P_{c}$, which also implies that the maximum $P_{c}$ achievable is ultimately limited by the covert constraint. Moreover, from the perspective of covertness, with the same value of $P_{c}$, the scheme with CJ outperforms the scheme with UJ and the scheme without a jammer in terms of Willie’s DEP. We also observe that the two curves for “Willie with CJ” intersect. A higher $r_{sd}$ results in a larger decoding threshold $\gamma_{th}$ for the public message. Consequently, Alice must use a large amount of power to transmit the public message in order to ensure successful decoding under both hypotheses $H_{0}$ and $H_{1}$. When $P_{c}$ is relatively low, the distinguishability between the total transmit powers under hypotheses $H_{0}$ and $H_{1}$, i.e., $P_{r,0}$ and $P_{r,1}$, is relatively small. As a result, the received power at Willie under $H_{0}$ and $H_{1}$ becomes more indistinguishable. It impairs Willie’s detection performance, leading to a higher detection error probability, which explains why the curve for larger $r_{sd}$ lies above the other. When $P_{c}$ gradually increases to a certain extent, the distinguishability of the received powers at Willie under $H_{0}$ and $H_{1}$ becomes greater, and the detection error probability of Willie gradually decreases, ultimately leading to the curve for larger $r_{sd}$ lying below the other.

**Figure 3:**
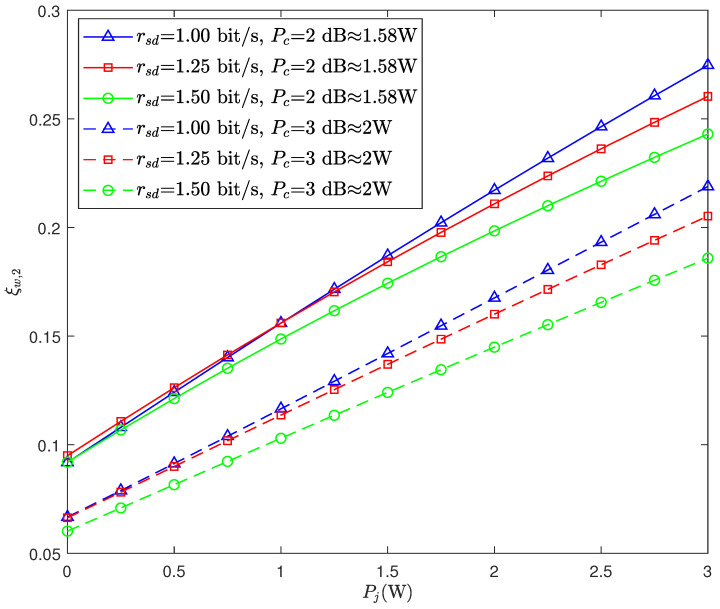
The DEP of Willie $\xi_{w,2}$ versus jamming power $P_{j}$ for different values of $r_{sd}$.

Figure 3 illustrates the impacts of $P_{j}$ and $r_{sd}$ on the DEP of Willie. As can be observed, $\xi_{w,2}$ increases monotonically as $P_{j}$ increases. This is because an increase in $P_{j}$ leads to higher $P_{r,0}$ and $P_{r,1}$. Thus, the signal strengths received by Willie under hypotheses $H_{0}$ and $H_{1}$ may have higher similarity, which can disrupt the detection of Willie and result in higher DEP. We can see that, with the same covert transmit power and the same jamming power, a higher value of $r_{sd}$ leads to a lower value of $\xi_{w,2}$, i.e., a high public message transmission rate will reduce the covertness.

**Figure 4:**
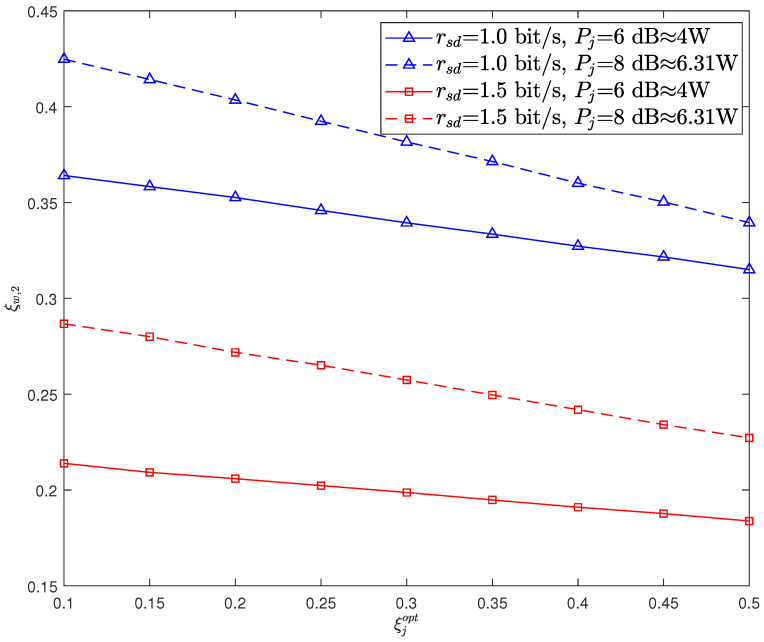
The DEP of Willie $\xi_{w,2}$ versus the DEP of CJ $\xi_{j}^{opt}$ with $P_{c}=$ 5 dB.

Figure 4 illustrates the relationships between $\xi_{w,2}$ and $\xi_{j}^{opt}$. It can be seen that $\xi_{w,2}$ decreases with the increase of $\xi_{j}^{opt}$, i.e., the covertness degrades as $\xi_{j}^{opt}$ increases. This is because a higher DEP of CJ can lead to a greater likelihood of its failure in its role as an effective jammer. Specifically, for $H_{0}$, CJ may have a higher probability of not transmitting jamming signals, and for $H_{1}$, CJ may more likely transmit jamming signals. Hence, a higher DEP of the CJ makes it more likely to perform incorrect behaviors, thereby improving the detection accuracy of Willie.

**Figure 5:**
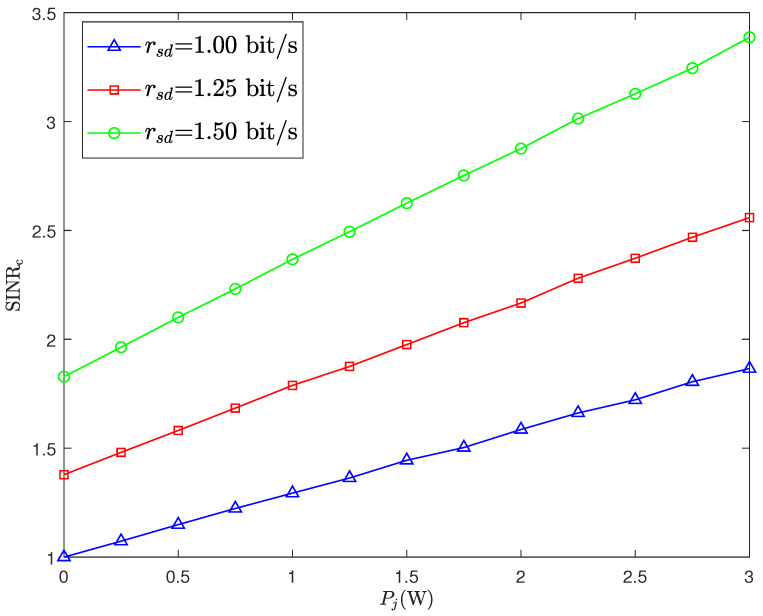
SINR at Bob ${\mathrm{SINR}}_{c}$ versus jamming power $P_{j}$.

Figure 5 illustrates the SINR at Bob versus $P_{j}$ for different $r_{sd}$. It can be observed that for a fixed $r_{sd}$, Bob’s SINR gradually increases with $P_{j}$. This is because, as the jamming power increases, Alice correspondingly raises its transmit power to overcome the interference. The effect of the power increment outweighs the loss caused by the interference, resulting in a slight improvement in the SINR. Meanwhile, it is also observed that when $P_{j}$ is fixed, Bob’s SINR increases with $r_{sd}$. The reason is that a higher $r_{sd}$ leads to a larger decoding threshold $\gamma_{th}$ for the public message, which in turn increases the transmit power allocated by Alice to the public message. As a result, Bob’s SINR increases. Furthermore, it also reveals that although increasing $P_{j}$ helps confuse Willie’s detection, it significantly raises Alice’s power consumption and may be limited by the maximum power constraint.

**Figure 6:**
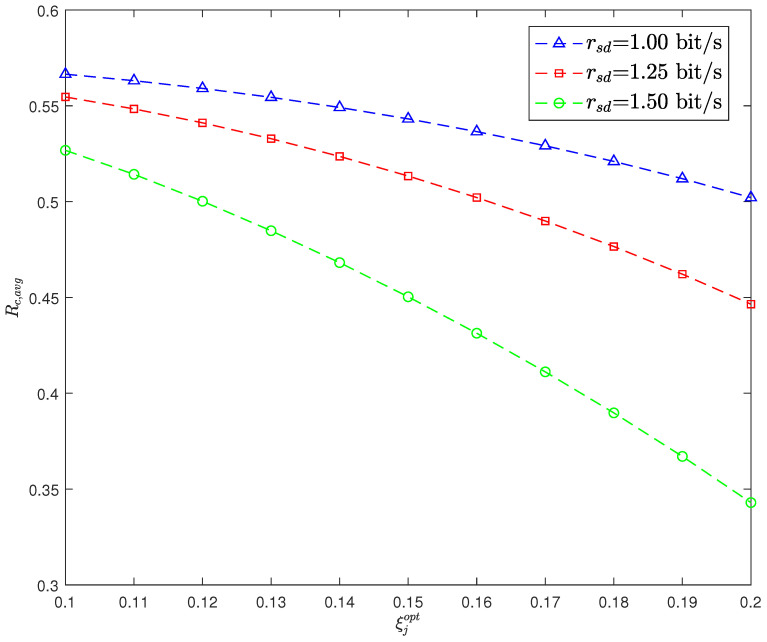
Average covert rate $R_{c,avg}$ versus the DEP of CJ $\xi_{j}^{opt}$ with $P_{c}=$ 2 dB.

Figure 6 illustrates the ACR $R_{c,avg}$ versus $\xi_{j}^{opt}$ for different $r_{sd}$. It can be observed that $R_{c,avg}$ decreases as $\xi_{j}^{opt}$ increases. This is because when Alice transmits its covert message to Bob, a higher DEP leads to the CJ being more likely to transmit unnecessary jamming, which reduces the SINR for decoding the covert message and thus degrades the ACR. According to (8), a lower value of $\gamma_{th}$, i.e., a lower value of $r_{sd}$, can increase the likelihood that the necessary condition for performing covert transmission is met, thereby enabling a higher ACR. Therefore, it can be seen that, with the same $\xi_{j}^{opt}$, $R_{c,avg}$ increases as $r_{sd}$ decreases.

## 4. Discussion

Our work aims to reveal the performance behavior of covert communication that is assisted by a CJ and embedded in a public communication system where channel uncertainty exists. Our research indicates that even though channel uncertainty impairs the detection performance of the CJ and thus has a negative impact on the covert performance, the application of the CJ can still effectively enhance the covert performance of the system, compared to the schemes with an uninformed jammer or without a jammer.

Moreover, our work reveals a fundamental relationship between the parameters of the public communication system and the achievable level of covertness. Specifically, we find that a higher public transmission rate can degrade the overall covertness of the system. Our analysis also identifies a key factor that positively influences covert performance: the detection accuracy of the cognitive jammer. The high detection accuracy of the CJ can contribute to enhancing performance in terms of both covertness and ACR.

## 5. Conclusions

In this work, we studied covert communication with a cognitive jammer embedded in a public communication system. We have derived the DEP for the cognitive jammer and warden, as well as the expression for the ACR. The impacts of system parameters on the system performance have been analyzed, and this analysis indicates that applying CJ to assist covert communication embedded in a public communication system can outperform the scheme with an uninformed jammer or without a jammer in terms of covertness. A high public transmission rate can degrade the covertness; however, high detection accuracy of the CJ can contribute to enhancing performance in terms of both covertness and ACR.

## Data Availability

The original contributions presented in this study are included in the article. Further inquiries can be directed to the corresponding author.

## Abbreviations

The following abbreviations are used in this manuscript:

CJ	Cognitive jammer
DEP	Detection error probability
ACR	Average covert rate
IoT	Internet of Things
LPD	Low probability of detection
MIMO	Multiple-input multiple-output
AWGN	Additive white Gaussian noise
UAVS	Unmanned aerial vehicles
SNR	Signal-to-noise ratio
AAV	Autonomous aerial vehicle
APF	Artificial potential field
CCI	Co-channel interference
RIS	Reconfigurable intelligent surface
SCA	Successive convex approximation
SDR	Semidefinite relaxation
CAoI	Covert age of information
BCA	Block coordinated ascent
SSP	System security probability
ISAC	Integrated sensing and communication
BCD	Block coordinate descent
STAR-RIS	Simultaneously transmitting and reflecting reconfigurable intelligent surface
QoS	Quality-of-service
CR	Cognitive ratio
CSI	Channel state information
UJ	Uninformed jammer
SINR	Signal-to-interference-plus-noise ratio
FA	False alarm
MD	Missed detection
